# A novel hybrid approach for “Scarless” (at the neck) lateral neck dissection for papillary thyroid carcinoma: A case series and literature review

**DOI:** 10.3389/fonc.2022.985761

**Published:** 2022-12-09

**Authors:** Zhen-Xin Chen, Jing-Bao Chen, Feng-Shun Pang, Zhan-Hong Lin, Xiao-Bo Zhang, Bei-Yuan Cai, Wei-Wu Zheng, Ying Cao, You Qin

**Affiliations:** Department of Minimally Invasive Surgery, The Second Affiliated Hospital of Guangzhou University of Chinese Medicine (Guangdong Provincial Hospital of TCM), Guangzhou, China

**Keywords:** lateral neck dissection, scarless, thyroid cancer, robotic, endoscopic

## Abstract

Lateral neck dissection (LND) is a necessary treatment for thyroid cancer with lateral lymph node metastasis. However, the defect created during open surgery leaves a visible scar on the neck. With advancements in surgical technology, many robotic and endoscopic surgical techniques have been reported as alternatives to open surgery. In this study, we present a case series demonstrating the successful application of a novel hybrid approach for endoscopic LND and a review of different surgical approaches for “scarless” (at the neck) LND. We performed endoscopic LND *via* a combined chest and transoral approach in 24 patients between January 2021 and March 2022. The surgery was completed successfully in all patients with an average operation time of 298.1 ± 72.9 min. The numbers of positive/retrieved lymph nodes at levels II, III-IV, and VI were 0.7 ± 0.9/8.4 ± 4.1, 3.6 ± 2.7/19.5 ± 6.8, and 4.9 ± 3.9/10.3 ± 4.5, respectively. Complications included transient hypoparathyroidism in 10 patients, transient recurrent laryngeal nerve injury in 1 patient, internal jugular vein (IJN) injury in 1 patient, IJN sacrifice due to cancer invasion in 1 patient, and chyle leak in 1 patient, and no cases of tumor recurrence were observed during follow-up. The present case series indicates that the combined chest and transoral approach is feasible and effective for performing LND. Our review of different approaches for “scarless” (at the neck) LND identified advantages and disadvantages for all techniques. Our novel approach has unique advantages, and thus, it can provide an ideal surgical procedure for specific papillary thyroid carcinoma patients.

## 1 Introduction

As the incidence of thyroid cancer has continued to rise in recent years, thyroid cancer ranks fifth among the most common malignant tumors in females in 2020 (1). Papillary thyroid carcinoma (PTC) is the most common subtype of thyroid cancer, and more than 30% of patients with PTC develop lateral lymph node (LN) metastasis and require lateral neck dissection (LND) (2–6). For this operation, open surgery provides direct exposure to the lateral compartment, thereby enabling surgeons to perform a safe and quick LND. However, the traditional open LND surgery requires the creation of a large incision in the neck that leaves a highly visible scar. This scar on the neck after open LND has been found to have a negative impact on patients’ quality of life (7, 8). To reduce the adverse effect of scarring on the neck appearance, an endoscopic-assisted approach was developed, which allowed the size of the neck incision to be greatly reduced (9). Since then, with technical advancements in minimally invasive surgery, many other approaches for “scarless” (at the neck) LND have been tested over the past decade. Depending on the surgical instruments employed, these techniques can be divided into robotic approaches and endoscopic approaches. According to the different access routes, the robotic approaches are subdivided into the transaxillary approach (TA), retroauricular approach (RA), bilateral axillary breast approach (BABA), transoral approach (TO), and transaxillary and retroauricular approach (TARA), whereas the endoscopic approaches are subdivided into the chest approach and transoral approach. However, all of the reported surgical approaches to date had limitations. For example, the high cost of robotic instruments has made robotic LND difficult to popularize. With the endoscopic LND approaches, due to obstruction by the clavicle, dissection of LNs at levels IV and VI *via* the chest approach is inherently challenging (10). Additionally, although the superior-to-inferior view of the transoral approach is helpful for LNs dissection at levels IV and VI, it does not permit complete dissection of LNs at level II.

In the present study, we developed and tested a new hybrid endoscopic approach for LND that combines the advantages of the chest approach and the transoral approach and is theoretically more conducive to complete dissection of LNs at levels II-IV and VI. In addition, we reviewed various approaches to “scarless” (at the neck) LND and considered the advantages and disadvantages of each approach. With many techniques available for LND, it is important to determine the most appropriate treatment that matches each patient’s needs.

## 2 Materials and methods

### 2.1 Patients

The present case series included all 24 patients who underwent endoscopic LND *via* the combined chest and transoral approach between January 2021 and March 2022 in our center. The study protocol was approved by the Institutional Review Board for Ethics at the Guangdong Provincial Hospital of Traditional Chinese Medicine. The inclusion criteria for this study were: confirmed diagnosis of PTC with lateral LNs metastasis (level II, III, or IV) and treated by endoscopic LND *via* the combined chest and transoral approach. The exclusion criteria were: 1) evidence of distant metastasis or metastatic LNs at level I or V; 2) invasion of surrounding tissues; 3) a history of surgery or radiotherapy at the neck; 4) max dimension of the thyroid cancer > 4 cm and 5) body mass index (BMI) > 40 kg/m2.

### 2.2 Operative procedures

**Figure 1:**
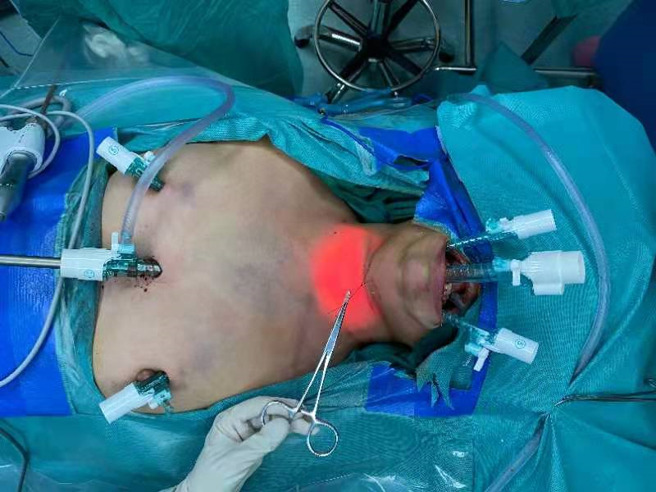
Representative image showing incision and trocar placement for endoscopic LND *via* the combined chest and transoral approach.

All patients were in supine position with their neck extended when an electromyogram (EMG) tube was used for endotracheal intubation. For all patients, the first prophylactic antibiotic (first-generation cephalosporin, cefazolin sodium 2 g) was administered 30 min before surgery. The first step of the operation was total thyroidectomy and LND *via* the chest approach. For this step, a parasternal incision was made at the nipple level, and two incisions were made at the 11 o’clock position of the left areola and 1 o’clock position of the right areola. A 10-mm trocar and two 5-mm trocars were inserted through parasternal incision and areola incisions, respectively (**Figure 1**). High-flow carbon-dioxide gas was insufflated at a pressure of 6 mmHg. A 30° laparoscope was inserted through the 10-mm trocar. After establishment of the initial working space, total thyroidectomy and endoscopic LND were performed in sequence. The detailed procedures for endoscopic LND were described in our previous study (11). It should be mentioned that LNs at level II-IV was routinely dissected. The spinal accessory nerve is used as a landmark to subdivide level II into IIb, the portion above and behind the nerve, and IIa, the portion that lays anteroinferiorly (12). During the operation, we explicitly dissected the spinal accessory nerve and completed dissection of LNs at level IIa and IIb. Next, three other trocars were inserted under the lower lip at the oral vestibular area, including a 10-mm trocar at the midline and two 5-mm trocars at the level of the first premolars (**Figure 1**). The central compartment was identified by the brachiocephalic trunk artery inferiorly, carotid artery laterally, and a deep layer of deep cervical fascia posteriorly. Careful attention was paid to dissecting and protecting the recurrent laryngeal nerve (RLN) until it descends into the chest. Then the LNs of level IV were observed, especially those between the sternocleidomastoid muscle (SCM) and sternohyoid muscle. Supplementary dissection of LNs at level IV was performed if necessary.

### 2.3 Postoperative management and follow−up

Patients resumed a normal diet 6 h after surgery, and the second prophylactic antibiotic (cefazolin sodium 2 g) was administered within 24 h after surgery. Once the drainage volume was <30 mL/day, the drainage tubes were removed. The numbers of retrieved and positive LNs, the operation time, and the postoperative hospital stay were recorded. Postoperative follow-up visits were scheduled at 1, 3, and 6 months and then annually thereafter. Hoarseness, cough, vocal cord function, parathyroid hormone level, color and volume of drainage fluid, limb lift restriction, other complications, and tumor recurrence were monitored during follow-up. Limb lift restriction was defined as debilitating shoulder droop and inability to raise the arm above the horizon (12). Hypoparathyroidism was defined when the level of PTH was lower than the lower limit (11 pg/mL) of the normal range of the hormone. The patients with hypoparathyroidism need enteral calcium supplementation. We recommend the use of calcium tablets, combined with calcitriol if necessary, to maintain normal calcium level.

### 2.4 Literature review

A literature review was performed using the keywords “neck dissection or lateral lymph node dissection” and “endoscopic or robot or robotic” to search titles in the PubMed. Only original English articles involving thyroid cancer patients were selected for review. Because none of the studies were randomized trials and several studies were missing important data, we did not perform an analysis of the combined results from the studies.

## 3 Results

This case series included 21 female patients and 3 male patients with a mean age of 34.9 ± 11.5 years and a mean body mass index (BMI) of 22.3 ± 3.7 kg/m^2^. In all patients, the combined chest and transoral approach for LND was performed successfully by the same surgeon, and no cases required conversion to open thyroidectomy. The mean operating time which represents - skin to skin was 298.1 ± 72.9 min, and the mean postoperative hospital stay was 5.8 ± 1.6 day. The mean numbers of positive/retrieved LNs from levels II, III-IV and VI were 0.7 ± 0.9/8.4 ± 4.1, 3.6 ± 2.7/19.5 ± 6.8 and 4.9 ± 3.9/10.3 ± 4.5, respectively.

Complications included transient hypoparathyroidism in 10 patients, transient RLN palsy in 1 patient, internal jugular vein (IJN) injury in 1 patient, IJN sacrifice due to cancer invasion in 1 patient, and chyle leak in 1 patient. All cases of transient hypoparathyroidism and RLN palsy resolved spontaneously within 3 months. No patients experienced permanent RLN palsy, permanent hypoparathyroidism, wound infection, limb lift restriction, tracheal fistula, seroma, hematoma or Horner’s syndrome. The mean follow-up period was 7.9 ± 4.9 months, and no cases developed tumor recurrence during follow-up (**Table 1**).

**Table 1:** Surgical outcomes of thyroid cancer patients who underwent robotic/endoscopic/open LND.

Variable	Robotic					Endoscopic			Open
	Kim et al (TA) (13)	Lira et al.(RA) (14)	He et al (BABA) (15)	Tae et al.(TO) (16)	Kim et al.(TARA) (17)	This study	Yan et al (Chest) (18)	Tan et al (TO) (19)	Kang et al (Open) (20)
Age	34.43±9.66	29.9	41.2±11.9	28	40.1	34.9±11.5	28.8±8.3	29.2±5.5	46.1±13.0
Sex(female/male)	394/106	11/1	197/63	1/0	17/5	21/3	149/6	19/1	83/26
BMI	22.37±3.39	23.4	23.9±3.7	NA	NA	22.3±3.7	NA	21.62	NA
Tumor size(cm)	1.33±0.78	NA	1.07±0.69	NA	1.17±0.68	1.8±1.1	1.26±0.71	1.12	1.49±0.80
Postoperative hospital stay(days)	5.47±1.42	3.4	6.5±2.6	NA	9.2	5.8±1.6	6.02±1.94	6.8±1.3	8±5.2
Operation time(mins)	293.71±67.22	340	201±63	295	209.4	298.1±72.9	278.2±38.6	146.0±18.7	218.2±43.8
No. of retrieved lymph nodes	42.99	27.8	17.9	41	40.24	38.2	22.91	18.3	39.4
II III-IV V	II-V 36.02	II-VI 27.8	II-V 17.9	Not dissected 29 Not dissected	10.36 16.09 6.68	8.4±4.1 19.5±6.8 Not dissected	8.23 14.68 Not dissected	Not dissected 10.9 Not dissected	II-V 31.0
VI	7.25		NA	12	7.1	10.3±4.5	NA	7.4	8.6
No. of positive lymph nodes	NA	NA	NA	7	7.55	9.2	3.18	4.7	NA
II III+IV V	NA NA NA	NA NA NA	NA NA NA	Not dissected 6 Not dissected	1.59 3.59 0.36	0.7±0.9 3.6±2.7 Not dissected	II-IV 3.18 Not dissected	Not dissected 2.7 Not dissected	NA NA NA
VI	NA	NA	NA	1	2	4.9±3.9	NA	2.0	NA
Complications									
Transient hypoparathyroidism	152(30.4%)	2(16.7%)	51(19.6%)	0	6(27.3%)	10(41.6%)	NA	0	50(45.9%)
Permanent hypoparathyroidism	20(4%)	0	0	0	0	0	NA	0	0
Transient RLN injury	20(4%)	3(25%)	3(1.15%)	0	2(9.1%)	1(4.2%)	8(5.2%)	1(5%)	3(2.8%)
Permanent RLN injury	5^a^(1%)	0	0	0	0	0	0	0	0
Internal jugular vein injury	0	0	0	0	0	1(4.2%)	19(12.3%)	0	0
Chyle leak	26(5.2%)	1(8.3%)	2(0.7%)	0	1(4.5%)	1(4.2%)	4(2.6%)	0	5(4.6%)
Internal jugular vein sacrifice due to cancer invasion	0	0	0	0	0	1(4.2%)	0	0	0
Tracheal fistula	0	0	1(0.4%)	0	0	0	0	0	0
Wound infection	1(0.2%)	1(8.3%)	1(0.4%)	0	0	0	2(1.3%)	0	1(0.9%)
Seroma	16(3.2%)	0	3(1.15%)	0	2(9.1%)	0	0	0	15(13.8%)
Hematoma	3(0.6%)	0	0	0	1(4.5%)	0	3(1.9%)	0	3(2.8%)
Horner’s syndrome	2(0.4%)	0	0	0	0	0	0	0	0
Limb lift restriction	0	0	0	0	0	0	6(3.9%)	0	0
Follow-up(months)	NA	NA	28.6±8.3	NA	15.9±5.2	7.9±4.9	NA	24.3±9.1	NA
Recurrence	5	0	1	0	0	0	2	0	0

a Including one patient whose the nerve was sacrificed due to cancer invasion.

NA, Not Available.

The characteristics and surgical outcomes of other cohorts reported in previous studies by different investigators also are listed in **Table 1** for comparison.

## 4 Discussion

Neck dissection of a malignancy was first described in 1906, and this surgery requires removal of all lymphatic and non-lymphatic structures between the platysma and the prevertebral fascia in the lateral neck, except for the common carotid artery and vital motor nerves. In 1980, a method for modified radical neck dissection was reported and then accepted as a substitute for classic neck dissection. The modified technique preserves one or more of the following structures: IJV, SCM, and spinal accessory nerve (21). Another method termed selective neck dissection refers to removal of less than all five nodal levels and is directed by the patterns of lymphatic drainage from the primary tumor, with preservation of the IJV, SCM, and spinal accessory nerve (12). Selective neck dissection is the most commonly used neck dissection surgery for the management of lateral neck metastasis of PTC, and an expert consensus in 2017 from China (only published in Chinese) suggested that comprehensive neck dissection of at least nodal levels IIA, III and IV should be performed when indicated to optimize disease control. Although the extent of the surgical dissection is decreased with this method, the required incision for this open surgery was similar in size to that required for traditional neck dissection (20, 22). Studies have documented that the change in neck appearance after open LND can negatively impact the quality of life of patients (7, 8). Therefore, surgeons have attempted to apply minimally invasive techniques to LND for treatment of PTC. As a result of these efforts, today robotic and endoscopic methods for LND as PTC treatment are offered in many hospitals worldwide. Considering the disadvantages of these methods, we developed a novel approach for endoscopic LND, and in this study, recorded the short-term clinical outcomes of patients in order to evaluate the feasibility of our approach. Upon reviewing all previously reported “scarless” (at the neck) LND methods for the treatment of thyroid cancer, we compared the advantages and disadvantages of the various approaches and the novel approach introduced in the present study.

### 4.1 Literature review of robotic LND techniques

Compare with endoscopic instruments, robotic instruments offer the following advantages: 1) movement with 7 degrees of freedom that is stabilized and not affected by tremor; 2) three-dimensional endoscopic view; 3) automatically controlled traction; and 4) optimized ergonomics (23, 24). The use of robotic instruments for robotic LND provides the following advantages: 1) the ability to achieve more accurate and elaborate dissection facilitates easier identification and preservation of the parathyroid gland, nerves, vessels, and lymphangion, lowering the risks of complications; and 2) improved exposure theoretically allows dissection of LNs at all levels (21). However, compared with endoscopic LND, robotic LND has the following limitations: 1) higher cost; 2) longer operative time; and 3) increased risk of neck pain and stiffness due to separation of the SCM (23). Beyond these general advantages and disadvantages of robotic LND, specific approaches to robotic LND have unique characteristics that can be beneficial in specific cases. Below we review the main robotic LND techniques described in the literature.

#### 4.1.1 Transaxillary approach (TA)

**Figure 2:**
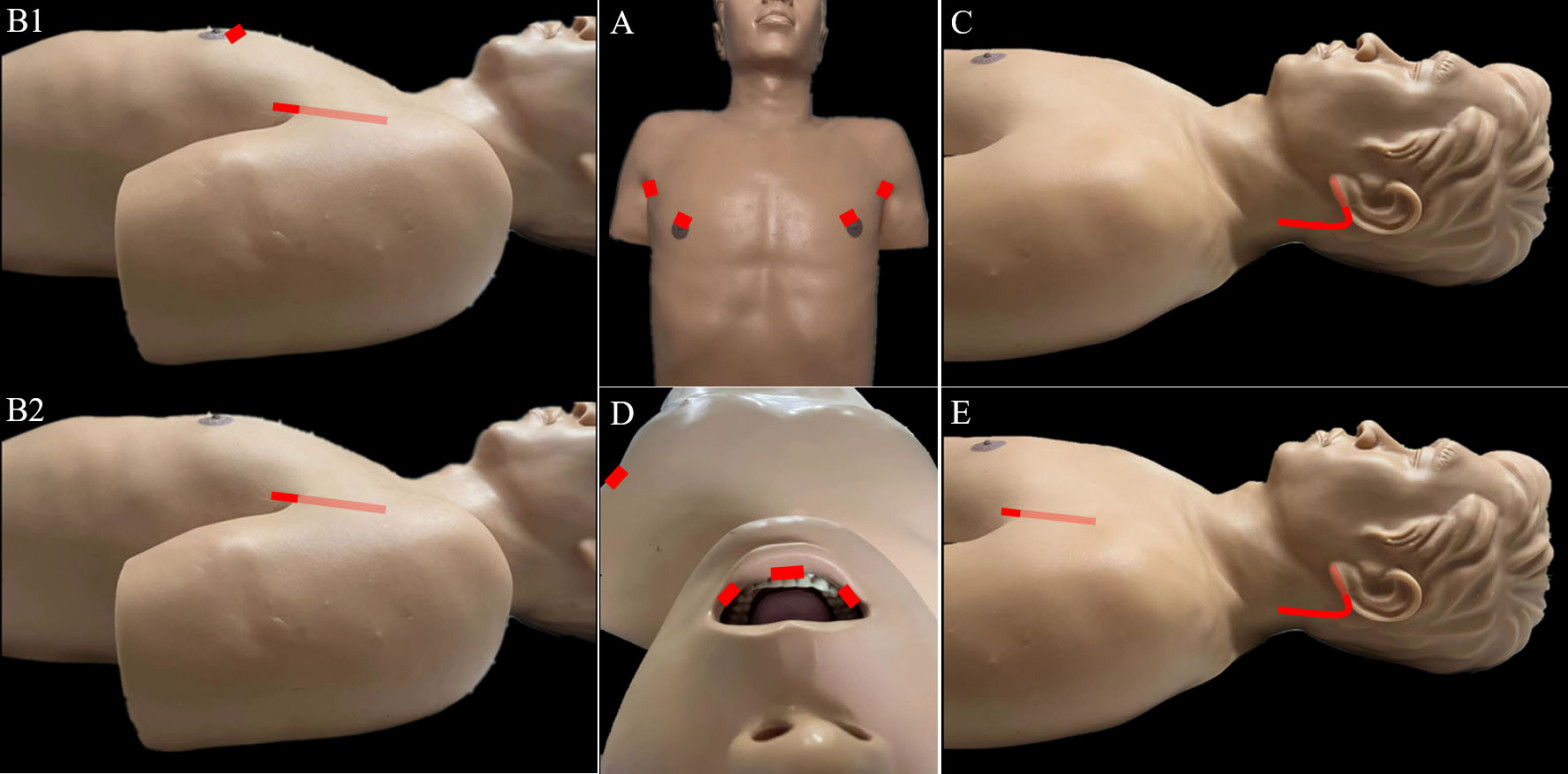
Incisions required for robotic LND *via* the: **(A)** bilateral axillary breast approach, **(B)** transaxillary approach, **(C)** retroauricular approach, **(D)** transoral approach, and **(E)** transaxillary and retroauricular approach.

The TA to robotic LND is also referred to as the gasless unilateral axillary (GUA) or gasless unilateral axillobreast (GUAB) approach. Notably, the transaxillary incision in GUA/GUAB approach was more lateral in the hair-bearing midaxillary line than the incision in the transaxillary approach used by Kang et al, which helped to improve the appearance by hiding the axillary scar completely under the arm (25). Robotic LND *via* the TA originally required two incisions, including a 7- to 8-cm vertical incision on the axilla and another incision on the breast in the case of GUAB (**Figure 2B1**). This approach was modified to the GUA approach, which involves only a single axillary incision without the breast incision, and all four robotic arms are inserted through this incision (**Figure 2B2**). The TA has been the most widely used method of robotic LND, with eight reports on this approach found in the literature, the first of which was published in 2010 (13, 20, 22, 25–29). In most studies, level IIA, III, IV and VB LNs were dissected *via* the TA. In addition, although LNs at levels IIB and VA are not routinely included in robotic LND, these LNs can also be exposed and dissected *via* this approach. In a report by Kim et al. (13), a cohort of 500 patients was treated *via* the TA. The average operating time was 293.71 min, and the average postoperative hospital stay was 5.47 days. Postoperative complications in their study included transient symptomatic hypocalcemia in 152 patients (30.4%), permanent hypocalcemia in 20 patients (4%), transient hoarseness in 20 patients (4%), permanent RLN injury in 5 patients (1%), RLN sacrifice due to cancer invasion in 1 patient (0.2%), seroma in 16 patients (3.2%), hematoma in 3 patients (0.6%), chyle leakage in 26 patients (0.8%), Horner’s syndrome in 2 patients (0.4%), wound infection in 1 patient (0.2%), and tumor recurrence in 5 patients (1%) **Table 1**). In studies comparing robotic LND approaches to open LND) (20, 26, 27), the reported oncologic and postoperative outcomes did not differ between robotic and open surgeries, and the robotic approaches were found to provide better cosmetic satisfaction among patients than open LND (**Table 2**).

**Table 2:** Summary of studies of robotic transaxillary LND.

Author	Study design	Cases	Operative time (min)	Complication profile (cases)	Postoperative hospitalstay (days)	Follow-up time(months)	Retrieved lymph nodes
Kang et al. (22)	Case series	33 (26F,7M)	280.8	Transient hypocalcemia 17 Transient hoarseness 2 Seroma 4 Chyle leak 3	5.4	14	Total (33.0) IIA (7.31); III (7.45) IV (8.17); Vb (6.34) Central (6.1);
Kang et al. (20)	Comparative	56 (46F,10M)	277.4	Transient hypocalcemia 27 Transient hoarseness 2 Seroma formation 5 Reoperation 1 Chyle leakage 5 RLN sacrifice d/t cancer invasion 1	3.92	NA	Total (37.3) IIA, III, IV, and Vb (31.1) Central (6.5)
Song et al. (25)	Case series	11^a^ (11F)	298.2	Chyle leakage 1 Transient Hypoparathyroidism 5 Recurrence 1	9.18^b^	38.0	Total (27.4) II (5.13); III (5.55) IV (7.1); Vb (3.44) Central (7.55);
Kim et al. (26)	Comparative	42 (33F,9M)	272.3	Seroma 4 Chyle leakage 4 RLN injury 3 Hypocalcemia (transient) 18 Arm movement disorder 1 Tumor recurrence 1	6.3	66	Total (35.2) IIA, III, IV, and Vb (30.5) Central (6.9)
Lee et al. (27)	Comparative	62 (57F,5M)	271.8	Transient Hypoparathyroidism 24 Transient RLN palsy 2 Wound problems 2 Transient chyle leakage 1	6.9	8.4	Total (38.0) IIA, III, IV, and Vb (32.8) Central (8.1)
Kim et al. (13)	Case series	500^c^ (394F,106M)	293.71	Transient hypocalcemia 152 Permanent hypocalcemia 20 Transient hoarseness 20 Permanent RLN injury 5 Seroma formation 16 Hematoma formation 3 Chyle leakage 26 Horner’s syndrome 2 Wound infection 1 Tumor recurrence 5	5.47	NA	Total (42.99) IIA, III, IV and V (36.02) Central (7.25)
Tae et al. (28)	Case series	12^d^ (12F)	310	Transient hypoparathyroidism 5 Seroma 3 Chyle leakage 1 Transient RLN palsy 1	7.3	12.9	Total (29.0) II (4.8);III (6.4); IV (7.7); V (3.9) Central (6.9);
Song et al. (29)	Comparative	25^e^ (24F,1M)	298^c^	Transient hypoparathyroidism 11 Transient vocal cord paralysis 1 Seroma 1 Chyle leakage 2 Tumor recurrence 1	8	29	Total (33.0) II (6.83); III (7.32); IV (8.68); V (4.47); Central (8.74);

^a^The overall study group comprised 21 patients, but 10 patients were not included in the review due to the use of charcoal suspension; ^b^total length of hospital stay; ^c^24 patients presented with recurrence and 30 cases performed bilateral LND; ^d^dissection of levels II-V was performed in 9 patients, dissection of levels II-IV was performed in 1 patient, and levels III-V was performed in 2 patients; ^e^dissection of levels II-V was performed in 14 patients, dissection of levels II-IV was performed in 4 patient, and levels III-V was performed in 7 patients.

NA, Not Available.

The reported advantages of the TA include: 1) a view similar to that of the open approach and relatively short operation time; and 2) safety and efficacy well-established by the literature (21, 30). The disadvantages of the TA include: 1) difficulty accessing level II LNs; 2) inability to perform contralateral LND; 3) difficulty of complete removal of level IV LNs due to interference by the clavicle; and 4) possible risks of anterior chest paresthesia and brachial plexus injury (21, 31).

#### 4.1.2 Retroauricular approach (RA)

For robotic LND *via* the RA, an L-shaped incision behind the auricle is needed, and three arms of the da Vinci Surgical System are typically used (**Figure 2C**). Two case series investigating the RA approach were found in the literature, and the first was published in 2014 (14, 32). In most cases, level II–V LNs were dissected *via* the RA, and exposure and dissection of the accessory nerve and LNs of levels II and III can be accomplished under direct vision using this approach. In the series of 12 patients reported by Lira et al. (14), complications included postoperative transient hypocalcemia in 2 patients (16.7%), transient vocal cord paresis in 3 patients (25%), surgical site infection in 1 patient (8.3%), and chyle leakage in 1 patient (8.3%) (**Table 3**) (14).

**Table 3:** Summary of studies of robotic retroauricular LND.

Author	Study design	Cases	Operative time (min)	Complication profile [cases]	Postoperative hospitalstay (days)	Follow-up time(months)	Retrieved lymph nodes
Byeon et al. (32)	Case series	4 (4F)	306.1	Hypoparathyroidism 2 Seroma 1 Chyle leakage 1	11	11.3	Total (43.0) II, III, IV and V (33.1); Central (9.8);
Lira et al. (14)	Case series	12 (1M, 11F)	340	Transient hypocalcemia 2 Transient vocal cord paresis 3 Surgical site infection 1 Reoperation for chyle leakage 1	3.4	NA	Total (27.8) II-VI (27.8)

NA, Not Available.

The noted advantages of the RA include: 1) reduced postoperative discomfort due to a relatively small dissection area; 2) ease of accessing level II; 3) suitability for obese patients; and 4) reduced risk of injury to great vessels, esophagus, and anterior chest sensory nerves (33, 34). The disadvantages of the RA include: 1) risk of injury to greater auricular and marginal mandibular nerves; 2) an opposite operative view compared with the open procedure; and 3) inability to perform contralateral LND (30, 34, 35).

#### 4.1.3 Bilateral axillary breast approach (BABA)

For robotic LND *via* the BABA, four incisions are needed, including a circumareolar incision in each breast and a vertical incision in each axilla (**Figure 2A**). The BABA has been a widely used method of robotic LND, with five reports investigating this approach found in the literature, the first of which was published in 2013 (15, 30, 36–38). In most cases, level II-V LNs were dissected *via* the BABA. Among the cohort of 260 patients studied by He et al. (15), the average operating time for the BABA was 201 min, and the average postoperative hospital stay was 6.5 days. Complications among this cohort included postoperative transient hypocalcemia in 51 patients (19.6%), transient hoarseness in 3 patients (1.2%), seroma in 3 patients (1.2%), chyle leakage in 2 patients (0.8%), tracheal injury in 1 patient (0.4%), wound infection in 1 patient (0.4%) and tumor recurrence in 1 patient (0.4%)(**Table 1**) . Notably, all 260 patients were extremely satisfied or satisfied with the cosmetic results of the BABA. From their comparative studies, Choi et al. (32) and Kim et al.[ (30, 36) concluded that robotic LND using the BABA is safe and provides oncologic and postoperative outcomes comparable to those of the open surgery (**Table 4**).

**Table 4:** Summary of studies of robotic BABA LND.

Author	Study design	Cases	Operative time (min)	Complication profile [cases]	Postoperative hospitalstay (days)	Follow-up time(months)	Retrieved lymph nodes
Choi et al. (30)	Comparative	12 (9F,3M)	277.08	Transient hypoparathyroidism 2 Transient vocal cord palsy 1	3.92	NA	Total (32.58) II (5.17); III (5.33); IV (7.75);V (2.92); VI (10.33);
Seup Kim et al. (36)	Comparative	13 (11F,2M)	382.3	Chyle leakage 1	5.4	13.2	Total (41.7) IIA, III, IV, Vb(28.9); Central (12.8);
He et al. (15)	Case series	260^a^ (197F,63M)	201	Transient hypocalcemia 51 Temporary vocal cord paresis 3 Seroma 3 Tracheal fistula 1 Wound infection 1 Chyle leakage 2 Tumor recurrence 1	6.5	28.6	Total (17.9) II-IV, Vb (17.9) Central (NA)
Yu et al. (37)	Case series	15 (14F,1M)	272.7	Transient hypocalcemia 7 Temporary vocal cord palsy 1 Horner’s syndrome 1	3.1	18.7	Total (28.8) II-V (20.7); Central (8.1);
Song et al. (38)	Case series	4^b^ (2F,2M)	533	pleural effusion 1	6.5	27.3	Total (54.5) Central and bilateral II-IV,Vb (54.5)

^a^21 cases performed bilateral LND; ^b^all 4 cases performed bilateral LND.

The advantages of the BABA approach include: 1) a symmetric view similar to that obtained with the open approach, and 2) the ability to perform bilateral LND (15, 30). The disadvantages of the BABA include: 1) difficultly performing level IV dissection due to an inferior-to-superior view; 2) difficulty accessing level II; and 3) risk of injury to anterior chest sensory nerves and brachial plexus (30).

#### 4.1.4 Transoral approach (TO)

For robotic LND *via* the TO, one central incision and two lateral incisions are made in the lower lip at the oral vestibular area, and another incision is made in the axilla for insertion of a third robotic instrument (**Figure 2D**). The TO has been described in only one case report published in 2020 (16). The operative time was 295 min, and no major postoperative complications occurred. Notably, no LNs of levels II and V were dissected in this case (**Table 5**).

**Table 5:** Summary of studies of robotic transoral LND.

Author	Study design	Cases	Operative time (min)	Complication profile [cases]	Postoperative hospitalstay (days)	Follow-up time(months)	Retrieved lymph nodes
Tae et al. (16)	Case report	1 (F)	295	None	NA	NA	Total (41) III and IV (29) Central (12)

NA, Not Available.

The apparent advantages of the TO include: 1) excellent access and exposure to the bilateral thyroid lobes, and 2) relative ease of dissection from the inferior side of level IV due to a superior-to-inferior view. The disadvantages of the TO include: 1) risk of mental nerve injury; 2) risk of infection and the need for postoperative antibiotics; 3) inability to completely remove level II and III LNs, making it oncologically unsafe; and 4) an operative view opposite to that of the open procedure (16).

#### 4.1.5 Transaxillary and retroauricular approach (TARA)

For robotic LND *via* the TARA, in addition to an L-shaped incision behind the auricle, another axillary incision is needed (**Figure 2E**). The TARA has been reported in one case series and one case report (17, 39), and level II–V LNs were dissected *via* this approach. Notably, exposure and dissection of the accessory nerve and LNs at levels II, VA, and upper level III can be accomplished under direct vision *via* the RA incision, and thyroidectomy, central neck dissection, and the dissection of LNs at levels III, VB, and IV can be achieved *via* the transaxillary approach. Among the 22 patients included in the case series (17), the average operating time was 209.4 min, and the average postoperative hospital stay was 9.2 days. Postoperative complications in this series included transient hypoparathyroidism in 6 patients (27.3%), seroma in 2 patients (9.1%), chyle leakage in 1 patient (4.5%), hematoma in 1 patient (4.5%), earlobe numbness in 6 patients (27.3%), and transient hoarseness in 2 patients (9.1%) (**Table 6**).

**Table 6:** Summary of studies of robotic TARA LND.

Author	Study design	Cases	Operative time (min)	Complication profile [cases]	Postoperative hospitalstay (days)	Follow-up time(months)	Retrieved lymph nodes
Kim et al. (17)	Comparative	22 (17F, 5M)	209.4	Transient hypoparathyroidism 6 Seroma 2 Chyle leakage 1 Hematoma 1 Earlobe numbness 6 Transient hoarseness 2	9.2^a^	15.9	Total (40.2) II (10.36), III (8.18), IV (7.91), V (6.68), Central (7.1);
Byeon et al. (39)	Case report	1 (1F)	142	None	8	NA	Total (16) III-IV (7), Central (9);

a Total length of hospital stay.

NA, Not Available.

The TARA offers the advantages of both the TA and RA approaches, and dissection of LNs at levels II–V can be achieved *via* this approach. The disadvantages of the TARA include: 1) the need for more incisions results in greater trauma; 2) risk of injury to the greater auricular nerve, marginal mandibular nerve, anterior chest sensory nerves, and brachial plexus; and 3) inability to perform contralateral LND.

### 4.2 Literature review of endoscopic LND techniques

Compared with robotic LND, endoscopic LND offers the following advantages: 1) much lower cost of endoscopic devices with the same effectiveness and safety as robotic devices for the treatment of thyroid cancer, and 2) endoscopic devices provide force feedback that robot surgery systems lack. The disadvantages of endoscopic LND include: 1) reduced versatility of the endoscopic devices compared with the robotic devices, which may lead to poor lateral compartment exposure, longer time under anesthesia, and a higher incidence of complications; 2) longitudinal separation of the SCM to expose the lateral compartment; and 3) restricted ability to dissect LNs at level V due to the lateral border of the SCM (11, 18, 23, 24).

#### 4.2.1 Chest approach

**Figure 3:**
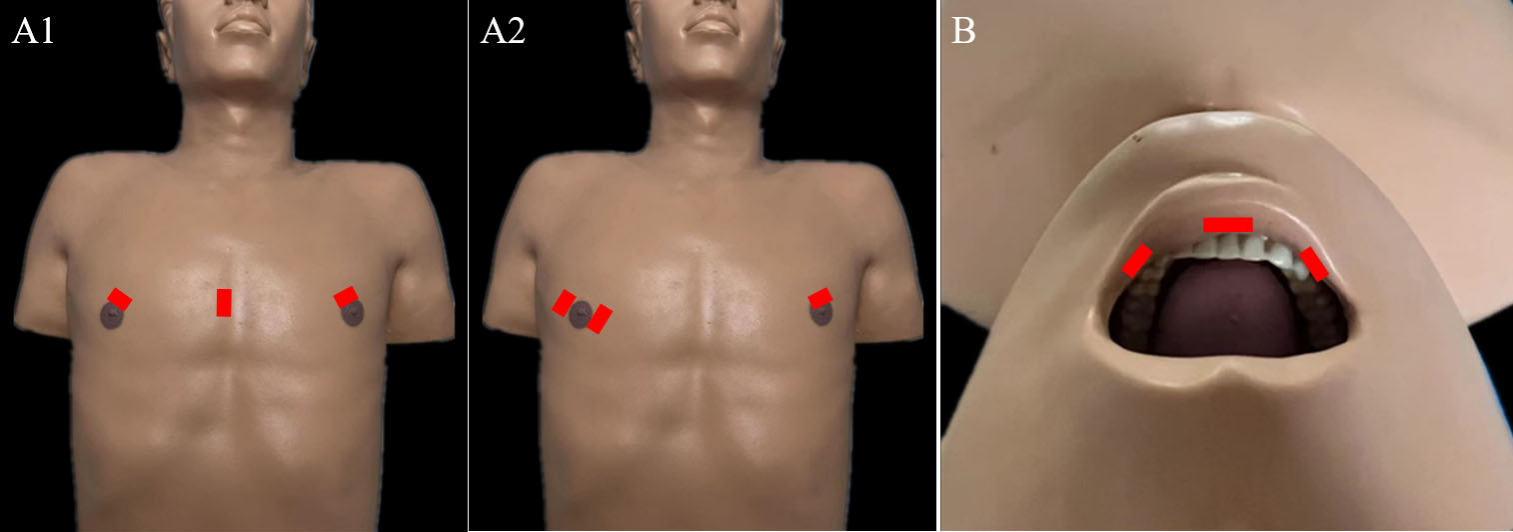
Incisions required for endoscopic LND *via* the: **(A)** chest approach and **(B)** transoral approach.

For endoscopic LND *via* the chest approach, three incisions are made, including a circumareolar incision in each breast and a third incision that can be either a parasternal incision (**Figure 3A1**) or circumareolar incision (**Figure 3A2**). The chest approach is the most widely used method of endoscopic LND. We found nine reports on this approach in the literature, the first of which was published in 2011 (11, 18, 40–46). Level II-IV LNs were routinely dissected in studies of endoscopic LND *via* the chest approach. Yan et al. (18) reported a cohort of 155 patients treated *via* the chest approach. Among this cohort, the average operating time was 278.2 min, and the average postoperative hospital stay was 6.02 days. Postoperative complications included transient hoarseness in 8 patients (5.2%), hematoma in 3 patients (1.9%), chyle leak in 4 patients (2.6%), infection in 2 patient (1.3%), IJN rupture in 19 patients (12.3%), limb lift restriction in 6 patients (3.9%), and local recurrence or residual tumor tissue in 2 patients (1.3%) (**Table 1**). In comparative studies of endoscopic LND *via* the chest approach versus open surgery, the reported oncologic and postoperative outcomes achieved with the chest approach were comparable to those achieved with open surgery (**Table 7**) (18, 42–44).

**Table 7:** Summary of studies of endoscopic LND via chest approach.

Author	Study design	Cases	Operative time (min)	Complication profile [cases]	Postoperative hospitalstay (days)	Follow-up time(months)	Retrieved lymph nodes
Yan et al. (18)	Comparative	155 (149F,6M)	278.2	Transit hoarseness 8 Hematoma formation 3 Chyle leak 4 Postoperation infection 2 Internal jugular vein rupture 19 Limb lift restriction 6 Local recurrence or residue at follow-up ultrasound 2	6.02	NA	Total (22.91) II (8.23); III-IV (14.68);
Li et al. (40)	Case series	11 (11F)	196.3	None	4.3	5.6	Total (25.7) III-IV (18.3); VI (7.4)
Yan et al. (41)	Case series	12 (12F)	243	Transient hypocalcemia 1 Internal jugular vein injury 1	5	NA	Total (21.8) II (6.7); III-IV (15.2)
Guo et al. (42)	Comparative	18 (16F,2M)	235	Lymphatic leakage 1 Temporary RLN injury 1 Temporary hypoparathyroidism 4 Large blood vessels injury 2	7.3^a^	NA	Total (27.7) II (5.8); III+IV (16.3); VI (5.6)
Lin et al. (43)	Comparative	31^b^ (20F,11M)	135	Transient hypoparathyroidism 1	NA	48	Total (27) II-IV (18); VI (9)
Huo et al. (44)	Comparative	12 (9F,3M)	276.25	Lymphatic leakage 1 Temporary hypoparathyroidism 2	NA	NA	Total (33.5) II-IV (21.2); Vb (5.8); VI (6.5)
Qu et al. (45)	Case series	24^c^ (22F,2M)	238.8	Temporary RLN palsy 2 Temporary hypoparathyroidism 4 Lymphatic leakage 2 Jugular vein injury 2 Lung metastasis 1	NA	>12	Total (21.8) II (5.9); III-IV (15.9);
Wang et al. (46)	Case series	37^d^ (35F,2M)	338.2	transient hypocalcemia 12 permanent hypocalcemia 1 transient RLN palsy 3 accessory nerves injury 2 Chyle leak 1 chest wall ecchymosis 1 skin burn 1 Local recurrence 1	5	24	Total (49.4) Lateral (33.5); Central (15.9)
Chen et al. (11)	Case series	35 (27F,8M)	307.5	Lymphatic leakage 1 Cervical plexus injury7 Accessory nerve injury 3 Hypoglossal nerve injury 1 Internal jugular vein injury 2	5.9	18.1	Total (32.2) II (8.8); III (6.1); IV (9.3); VI (8.0)

^a^drainage time; ^b^2 patients underwent bilateral LND and their dates were not included; ^c^levels III-IV dissection had been performed in 6 patients and levels II-IV dissection had been performed in 18 patients; ^d^22 cases underwent level II-IV LND, 13 underwent level II-VI and partial level V, 1 patient underwent bilateral LND (level II-VI and partial level V), 1 patient underwent levels III-IV LND.

NA, Not Available.

The advantages of the chest approach for endoscopic LND include: 1) a symmetric view similar to that achieved with the open approach; 2) ability to perform bilateral LND; and 3) relative ease of level II LN dissection due to endoscopic magnification and less space restriction. The disadvantages of the endoscopic chest approach include: 1) restricted access for level IV dissections due to the clavicle, and 2) risk of anterior chest paresthesia.

#### 4.2.2 Transoral approach

For endoscopic LND *via* the transoral approach, three incisions are needed, including an incision at the midline of the lower lip and two incisions at the level of the first premolars (**Figure 3B**). Notably, LNs at levels II and V have not been dissected *via* this approach in the reported studies. The transoral approach is a less reported method of endoscopic LND compared with the chest approach, with only one case series and one case report found in the current literature (19, 47). Tan et al. (19) reported a cohort of 20 patients treated with endoscopic LND *via* the transoral approach. Among their cohort, the average operating time was 146 min, and the average postoperative hospital stay was 6.8 days. Postoperative complications included transient unilateral RLN palsy in 1 patient (5%) and effusion in the operative field in 2 patients (10%) (**Table 8**).

**Table 8:** Summary of studies of endoscopic LND via transoral approach.

Author	Study design	Cases	Operative time (min)	Complication profile [cases]	Postoperative hospitalstay (days)	Follow-up time(months)	Retrieved lymph nodes
Tan et al. (19)	Case series	20 (19F, 1M)	146	Transient RLN palsy 1 Effusion in the operative area 2	6.8	24.3	Total (18.3) III-IV (10.9); Central (7.4)
Ngo et al. (47)	Case report	1 (1F)	170	None	NA	NA	Total (15) II- IV (8); Central (7)

NA, Not Available.

The advantages and disadvantages of the transoral approach to endoscopic LND are similar to those of the robotic transoral approach.

#### 4.2.3 Combined chest and transoral approach

The endoscopic LND approach reported in the present study seeks to combine the advantages of the chest and transoral approaches to endoscopic LND. With this new approach, dissection of LNs at levels II-IV was first performed *via* the chest approach. Then supplementary dissection of the inferior side of level IV was performed *via* the transoral approach. As a result, this approach afforded completely unrestricted dissection of LNs at levels II-IV *via* endoscopic surgery. The disadvantages of our combined approach include: 1) greater trauma due to the requirement of more incisions, and 2) potential risks of prolonged operation time, mental nerve injury, infection, and anterior chest paresthesia. However, among the 24 patients in the present study, the hospital stay was comparable with that in a study of the chest approach for endoscopic LND (48) **Table 1**) which suggests that the addition of the small incision in the lower lip does not significantly increase the patient’s trauma. The operating time for the combined chest and transoral approach in this study was slightly longer than that for the chest approach, and this is attributed to the fact that once the initial operating space is established *via* the chest approach, no additional time is required for flap separation *via* the transoral approach. In addition, no patients developed with mental nerve injury or infection. Consistently, in previous studies of the transoral endoscopic thyroidectomy vestibular approach (TOETVA) technique, postoperative infection was rare, and the risk of mental nerve injury was low (49–51). Notably, the number of central LNs retrieved using our approach was greater than that achieved in the study by Kim et al. (13), and the number of lateral LNs retrieved *via* our combined chest and transoral approach was greater than that achieved *via* the chest approach (48) (**Table 1**). These advantages in LN dissection may be due to the benefits of the transoral approach for dissection of central LNs and supplementary LN dissection from the inferior side of level IV.

### 4.3 Limitations of the present study

Some limitations of our study should be acknowledged. First, this was a retrospective study and lacked of control group. The safety of the combined chest and transoral approach for endoscopic LND should be evaluated in prospective randomized controlled trials in the future. Additionally, the mean follow-up period in our cohort was only 7.9 ± 4.9 months, which is too short to draw conclusions on the effectiveness of our approach for the treatment of PTC.

## 5 Conclusions

Many different approaches for “scarless” (at the neck) LND have been reported, and each approach has a unique set of advantages and disadvantages. Surgeons need to be familiar with the advantages and disadvantages of each approach in order to recommend appropriate approaches for patients according to the individual characteristics of patients and tumors as well as the experience of the surgeon. The combined chest and transoral approach introduced in this study offers advantages of both the chest approach and transoral approach, potentially allowing more extensive dissection of LNs and achieving better oncological results. Once the safety of this approach is confirmed in a larger cohort, this novel approach may provide the most ideal surgical method for select patients.

## Data availability statement

The raw data supporting the conclusions of this article will be made available by the authors, without undue reservation.

## Ethics statement

The studies involving human participants were reviewed and approved by Institutional Review Board for Ethics at the Guangdong Provincial Hospital of Traditional Chinese Medicine. Written informed consent for participation was not required for this study in accordance with the national legislation and the institutional requirements.

## Author contributions

Z-XC, J-BC, F-SP, and YQ designed the concept of this study. Z-HL, X-BZ, B-YC, W-WZ, and YC collected the datasets. Z-XC, J-BC, and F-SP analyzed the data and wrote the manuscript. Z-XC and YQ revised the manuscript. All authors have read and approved the manuscript.
